# Self-Conscious or Fear of Hurting Another’s Feeling? An Experimental Investigation on Promise-Keeping

**DOI:** 10.3389/fpsyg.2020.576824

**Published:** 2020-09-30

**Authors:** Wenjie Zhang, Xianchen Zhu, Hongyu Guan, Tao Li

**Affiliations:** ^1^School of Economics and Management, Nanjing University of Science and Technology, Nanjing, China; ^2^Center for Experimental Economics in Education, Shaanxi Normal University, Xi’an, China

**Keywords:** promise, internalized norm mechanism, expectation-based mechanism, guilt aversion, trust game

## Abstract

We conducted an experimental investigation into whether the effect of non-binding verbal promises in enhancing cooperation among promisors is derived from the internalized norm mechanism or the expectation-based mechanism. We proposed a new experimental design based on the standard trust game to separate the two possible influence mechanisms of promises and assess the empirical support for these two mechanisms for the effect of promises. We also identified individuals’ cooperation preferences to further investigate whether the effect of promises and its underlying mechanism differ between individuals with different preferences. The results show that promises significantly improve promisors’ cooperation level, and this effect is only in line with the internalized norm mechanism rather than with the expectation-based mechanism. Additionally, the introduction of non-binding promises has different impacts on the behavior of selfish individuals and conditional cooperators, but both sets of the impacts can be interpreted as evidence for the internalized norm mechanism, while neither is supportive of the expectation-based mechanism. This paper provides empirical evidence consistent with the internalized norm mechanism for the effect of promises in promoting cooperation. There appears to be no support for the expectation-based mechanism.

## Introduction

Many theoretical and empirical studies have demonstrated that communication can promote trust and cooperation among individuals, and verbal promises play a critical role in it (Ostrom et al., 1992; Sally, 1995; Balliet, 2010). Thus, why do non-binding promises influence promisors’ behavior? In this regard, researchers have mainly proposed two possible influence mechanisms. The first possible influence mechanism is the internalized norm mechanism. The substance of this mechanism is that making promises activates promisors’ internalized norm of promise-keeping and a desire to comply with the norm of promise-keeping *per se* motivates individuals to keep their word and improve their level of cooperation (Kerr et al., 1997; Ellingsen and Johannesson, 2004; Vanberg, 2008; Ellingsen et al., 2010; Kawagoe and Narita, 2014; Krupka et al., 2016, etc.). That is, individuals keep their promises due to self-consciousness^1^. The second is the expectation-based mechanism which is on the basis of the theory of guilt aversion: a promise may change the expectation of the promisee, and the promisor chooses to keep his or her promise and improve the level of cooperation to avoid the feeling of guilt from not fulfilling the other party’s expectation (Dufwenberg and Gneezy, 2000; Charness and Dufwenberg, 2006; Amdur and Schmick, 2013; Battigalli et al., 2013; Ederer and Stremitzer, 2017, etc.). In other words, an individual keeps his or her promise due to the fear of hurting others’ feelings.

However, much debate still remains regarding how to distinguish between the two influence mechanisms of non-binding verbal promises and the differences in their explanatory power. Ismayilov and Potters (2016) and Schwartz et al. (2019) allude to the problems when they distinguish between and test the internalized norm mechanism and the expectation-based mechanism within the same experimental framework.

Referring to the basic experimental setting of Charness and Dufwenberg (2006), Ismayilov and Potters (2016) use a binary trust game with pre-play communication^2^. The key difference from Charness and Dufwenberg (2006) is that only half of the pre-play messages by trustees are delivered to trustors in Ismayilov and Potters’ experiment, and the other half of the messages are intercepted and replaced with blank paper^3^. Trustees know whether their own message is delivered or not before making decisions. Their experimental results show that the cooperation level of trustees who make a promise is significantly higher than that of trustees who do not make a promise even when messages are not delivered, which is a finding consistent with the norm-based explanation for promise-keeping. However, Ismayilov and Potters (2016) also indicate the endogeneity problem in their experimental results. By conducting a second control experiment that restricts the content of the communication, where promises are not permitted, they find that the average cooperation level of trustees in the second experiment is not lower, and is actually a little higher, than that in the first experiment where trustees can make promises. Therefore, Ismayilov and Potters (2016) conclude that the higher cooperation level of trustees who make a promise in the first experiment is due to self-selection, that is, trustees who make a promise are more trustworthy *per se*, and promises do not play a role in it. Nonetheless, they do not completely deny the internalized norm mechanism because of the existence of the endogeneity problem.

Following up on Ismayilov and Potters (2016), Schwartz et al. (2019) conduct a similar experiment. Differences in their experimental setting from Ismayilov and Potters’ are as follows. First, they extend the binary decision-making of trustees (cooperate or not cooperate) in Ismayilov and Potters’ experiment to trustees’ return decision, which is in the form of consecutive integers. Second, Schwartz et al. (2019) use the strategy method to acquire trustees’ return decisions, that is, trustees need to decide the amount they return in the case of their message being delivered and in the case of their message not being delivered, without knowing the actual delivery status of their message. Their experimental results show that the return level of trustees who make a promise in the communication stage is significantly higher when the promise is delivered than when the promise is not delivered, under which circumstance the return level of trustees is as low as that of trustees who do not make a promise. That is, Schwartz et al. (2019) find no support for the internalized norm mechanism and they only support the expectation-based explanation for promise-keeping.

Based on the aforementioned discussion, we find that, regarding the explanatory power of the internalized norm mechanism and the expectation-based mechanism for the influence of promises, Ismayilov and Potters (2016) and Schwartz et al. (2019) have found inconsistent evidence under a similar experimental structure (Table 1 provides a comparison of the two experiments). The results, thus far, are still somewhat mixed. Therefore, the experimental settings and results of the aforementioned two studies must be further discussed. Why is the result of Ismayilov and Potters’ experiment consistent with the internalized norm mechanism, while Schwartz et al. (2019) find no evidence for the internalized norm mechanism and only support the expectation-based mechanism under a similar experimental framework? We believe the possible reasons are as follows.

**TABLE 1:** Comparison between the experiments of Ismayilov and Potters (2016) and Schwartz et al. (2019).

Study	Treatment	Message status	Bs’ cooperation level		Conclusion	
			Promise	Non-promise	Expectation-based	Norm-based
Ismayilov and Potters, 2016	binary trust game	delivered	54%	42%	×	√
		undelivered	35%	14%		
Schwartz et al., 2019	trust game with continuous return	delivered	7.38	5.11	√	×
		undelivered	4.59	5.67		

First, Schwartz et al. (2019) have not addressed the endogeneity problem, which is the key issue in Ismayilov and Potters (2016)’s experiment; thus, the robustness of their experimental results remains up for discussion. Second, we posit that the strategy method used by Schwartz et al. (2019) may confuse the participants. In their experiment, trustees need to decide the amount they return conditional on the message being delivered and conditional on the message not being delivered simultaneously, and in this situation, trustees who make a promise are likely to interpret the difference between “promise being delivered” and “promise not being delivered” that the authors originally intended to investigate as the difference between “promise being delivered” and “no promise.” Thus, the return level of trustees under the condition of “promise not being delivered” is relatively low, leading to the difference in the conclusions from Ismayilov and Potters (2016). Finally, both of the aforementioned studies use free-form communication to investigate the effect of promises, and this may generate a substantial difference in the content of communication among different groups (Cadsby et al., 2015). Additionally, identifying and coding the communication messages mainly depend on experimenters’ subjective judgment, and experimental results may, therefore, be mixed with other interference.

The aim of this paper is then to present a new experimental design and attempt to address the aforementioned possible problems in the experiment of Ismayilov and Potters (2016) and Schwartz et al. (2019) to distinguish the internalized norm mechanism from the expectation-based mechanism for the effect of promises and provide reliable empirical evidence for the two mechanisms.

First, to control for the endogeneity problem, we adopt a within-subject design through which we can exclude the impact of personal traits when comparing individual behavior across treatments. Second, to avoid misunderstanding among participants, we emphasize the concept of *self-promise* in our experiment by directly informing trustees that their promise will be unknown to the corresponding trustor (similar to the setting of “undelivered promise”) and explicitly compare *self-promise* with *public promise* (similar to “delivered promise”) by setting two separate treatments. Third, we restrict the form of trustees’ promises to ensure the consistency of the content of communication among groups and the validity of promises (detailed experimental settings are provided in this paper).

Specifically, we introduce two forms of promises into the trust game and design three independent treatments, namely, the *no promise* treatment (T_1_), *self-promise* treatment (T_2_), and *public promise* treatment (T_3_), to conduct a cross-treatment comparison of an individual’s return behavior and investigate the effect of promises on it and the underlying mechanism of this effect. In the *public promise* treatment, promises play a dual role of activating trustees’ internalized norm of promise-keeping and changing trustees’ belief about the expectation of the corresponding trustor (second-order belief). Therefore, simply comparing the return level of trustees in the *public promise* treatment with that in the *no promise* treatment cannot help us differentiate these two influence mechanisms. Notably, in the *self-promise* treatment, making promises activates trustees’ internalized norm of promise-keeping but does not influences trustees’ second-order belief. Thus, if the return level of trustees in the *self-promise* treatment significantly increases compared with that in the *no promise* treatment, the results support the internalized norm mechanism. On this basis, we then compare the return level of trustees in the *public promise* treatment with that in the *self-promise* treatment to judge whether our experimental results are consistent with the expectation-based mechanism. To be more specific, (1) if there is no significant difference between the return level of trustees in the *public promise* treatment and that in the *self-promise* treatment, but both are significantly higher than the return level in the *no promise* treatment, only the internalized norm mechanism works; (2) if the return level of trustees in the *public promise* treatment is significantly higher than that in the *self-promise* treatment, but there is no significant difference in the return level of trustees between the *self-promise* treatment and the *no promise* treatment, only the expectation-based mechanism works; (3) if the return level of trustees in the *public promise* treatment is significantly higher than that in the *self-promise* treatment, and the return level in the *self-promise* treatment is significantly higher than that in the *no promise* treatment, both of the two mechanisms play a role.

Our experimental results show that the return level of trustees in the *self-promise* treatment is significantly higher than that in the *no promise* treatment, and there is no significant difference between the return level in the *public promise* treatment and that in the *self-promise* treatment. These results are in line with the internalized norm mechanism but do not support the expectation-based mechanism. Thus, we reach the same conclusion as Ismayilov and Potters (2016) in this paper: we both find evidence that supports the norm-based explanation for promise-keeping. Our results obviously differ from those in Schwartz et al. (2019), whose results are inconsistent with the internalized norm mechanism and support only the expectation-based mechanism.

Additionally, López-Pérez (2012) introduces individual heterogeneity into their norm-breaking aversion model and asserts that the effect of communication on an individual’s cooperation level is influenced by the type of individual’s other-regarding preference. Andrighetto et al. (2015) also point out that different individuals may be motivated by different concerns when making decisions. Therefore, we introduce individual preferences into our analysis. Referring to the experiment of Fischbacher et al. (2001), we adopt the strategy method in the trust game to identify each individual’s preference type and further investigate whether the influence of promises on trustees’ return decision and the influence mechanism behind this will be affected by individual preferences.

Our experimental results show that for selfish individuals, their average return levels in different treatments are all relatively low; however, their return level in the *self-promise* treatment remains significantly higher than that in the *no promise* treatment, and their return level in the *public promise* treatment is instead slightly lower than that in the *no promise* treatment. This finding means that even if selfish individuals attach more importance to their material outcomes, the impact of internalized norms remains significant. Therefore, the effect of promises on selfish individuals’ behavior supports the internalized norm mechanism, and there is no support for the expectation-based mechanism. For conditional cooperators, their return level in the *self-promise* treatment is significantly higher than that in the *no promise* treatment, and there is no significant difference in the return level of this type of trustees between the *public promise* treatment and the *self-promise* treatment. Thus, the effect of promises on conditional cooperators’ return decision is still only consistent with the internalized norm mechanism, not with the expectation-based mechanism^4^.

## Materials and Methods

### Participants

Our experiment was conducted at the lab center of Nanjing University of Science and Technology in China. Sixty undergraduates (24 from Economics) were recruited online, with 24 males and 36 females. The ages of the participants were between 18 and 21 and their average was 19. Two sessions were conducted with 32 participants in the first session and 28 participants in the second session. All participants were required to read and approve the written informed consent before participating in this experiment. The experiment was programmed and conducted with the software z-Tree (Fischbacher, 2007). The duration of the experiment was approximately 1 h, and the average payoff of the participants was RMB 26 (including a show-up fee of RMB 5).

### Design and Hypotheses

Our experimental treatments are based on the standard trust game of Berg et al. (1995). Two participants are randomly matched as a group, and each participant is randomly assigned either the role of the “trustor” (labeled as “A”) or the role of the “trustee” (labeled as “B”). Trustor A has an initial endowment of *E* tokens and decides to transfer *S*, which is within the interval [0, *E*], to trustee B. The amount received by trustee B is tripled, and then B chooses to return any amount *R*, which is restricted in the interval [0, 3*S*], to trustor A. The payoff of trustor A is *E*−*S* + *R*, and the payoff of trustee B is 3*S*−*R*.

In order to assess the empirical support for the internalized norm mechanism and the expectation-based mechanism, we have designed three separate treatments: *no promise* (T_1_), *self-promise* (T_2_), and *public promise* (T_3_).

The *no promise* treatment (T_1_) is the same as the standard trust game depicted above. The initial endowment of trustor A is 100 tokens.

In the *self-promise* treatment (T_2_), we introduce a promise-making stage into the standard trust game. Specifically, before trustor A decides how many tokens to transfer, trustee B should first make a promise about how much he or she will return. Referring to the strategy method employed in Fischbacher et al. (2001), we ask trustee B to fill out a conditional promise table, promising the ratio of the tokens, denoted as *r*, he or she will return as per all the possible amounts trustor A may transfer^5^. Trustee B’s promise will be unknown to the corresponding trustor A in this treatment, which is explicitly informed to all the participants. After the promise-making stage, trustor A, who is unaware of trustee B’s promise, decides the amount to transfer. The amount received by trustee B is tripled, and then trustee B chooses to return any amount of tokens to trustor A. Notably, promises in our experiment have the property of cheap talk; trustees do not incur punishment and pecuniary loss from not keeping their promises. It’s worth noting that we do not employ free-form communication as Ismayilov and Potters (2016) and Schwartz et al. (2019) do in the promise-making stage. In their experiment, trustees have an opportunity to send a message with any content except self-identifying information to the trustor, resulting in a substantial difference in the content of communication among different groups. Additionally, ex post identifying and coding of the messages (judging whether the message contains a promise or not) mainly depends on experimenters’ subjective judgment and different classifications may generate different conclusions. Therefore, we restrict the form of trustees’ promises in our experiment to ensure the consistency of the content of communication among groups and the validity of promises^6^.

**FIGURE 1:**
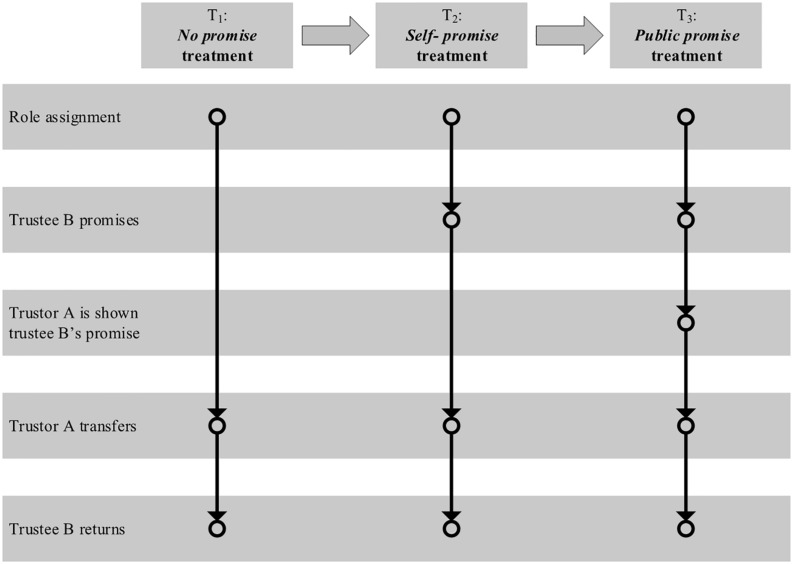
Timeline of the *no promise* treatment, *self-promise* treatment, and *public promise* treatment.

The *public promise* treatment (T_3_) is identical to the *self-promise* treatment (T_2_) except that trustee B’s promise will be shown to the corresponding trustor A after the promise-making stage and then trustor A decides how many tokens to transfer in T_3_. That is, in T_2_, trustee B makes a promise to himself or herself; in T_3_, trustee B makes a public promise to the corresponding trustor A. Figure 1 summarizes the timeline of the three main treatments.

We implement a within-subject design to control for the self-selection effect that exists in the experimental results of Ismayilov and Potters (2016). In our experiment, all the subjects participate in the *no promise* treatment, *self-promise* treatment, and *public promise* treatment in sequence. With such a design, we can exclude the impact of individual heterogeneity when comparing individual behavior across treatments and avoid the endogeneity problem resulting from the difference in each individual’s level of trustworthiness *per se*. Notably, each time participants enter a new treatment, the program will randomly rematch two anonymous participants as a new group; thus, there is no repeated interaction between any two participants across treatments, which reduces the possibility of the learning effect. Besides, if there is a learning effect, the main possible consequence is that both the amount transferred by trustor A and the amount returned by trustee B significantly decrease in the last treatment (i.e., the *public promise* treatment), which is obviously not the case in our experimental results. Therefore, we consider it less likely that a learning effect exists in our experiment. There is a fixed order of the three main treatments. To avoid the possibility that participants’ decisions in the last treatment are affected by their payoffs in the former two treatments, we do not provide participants with information about their payoffs in each treatment until the end of the experiment. That is, participants are informed of their payoffs in each treatment after all the decisions are made.

Each of the three main treatments comprises two rounds: in the first round, participants are assigned either the role of trustor A or the role of trustee B and make corresponding decisions, and then, without showing participants their payoffs in the first round, the program will exchange the role of the two participants within a group and repeat the procedure of the first round. Through this setup, we can obtain the decisions of all 60 participants both as a trustor and as a trustee. However, in the experimental instruction and interface, participants are informed that the program will randomly rematch two participants as a group and assign the roles to reduce the strategic consideration between participants from repeated interactions while ensuring the number of observations^7^. To avoid the wealth-accumulation effect, the program will randomly select one of the two rounds for payoff calculation and determine the actual payoffs of participants in the treatment.

Besides, considering the effect of heterogeneous preferences on individual behavior (Camerer, 2003; Lundquist et al., 2009; Cartwright, 2018), we implement a *preference identification* treatment (T_0_) at the beginning of the whole experiment to elicit each participant’s cooperation preference in the context of the trust game, further investigating whether the effect of *self-promise* and *public promise* will differ among individuals with different preferences. Specifically, referring to the strategic method of identifying individual preferences in Fischbacher et al. (2001), we make all the participants first play the role of trustee B and ask them to decide how many tokens to return as per all the possible amounts trustor A may transfer, namely, conditional return. Different from the aforementioned three main treatments, in the *preference identification* treatment (T_0_), the initial endowment of trustor A is 20 tokens. Thus, the non-zero amount trustor A may transfer are integers that lie in the interval [1, 20], which is tripled on the way to trustee B. In this circumstance, we can observe how participants adjust the amount they return according to the amount the other party transfers and then identify the type of their preferences. To ensure participants take their decisions seriously, we then make all the participants also play the role of trustor A and decide the amount to transfer, before knowing which role they will actually be assigned. Once all the participants have made their decisions both as trustor A and as trustee B, the program randomly matches two participants as a group, assigns the roles, and determines the payoffs in the *preference identification* treatment based on the decision each of the two participants has made for the role actually assigned.

With this design, we can obtain each participant’s return decisions as trustee B in the *no promise* treatment, *self-promise* treatment, and *public promise* treatment. Our aim is to assess whether the effect of promises on trustees’ cooperation level is derived from the internalized norm mechanism, which emphasizes the role of promises in activating trustees’ internalized norm of promise-keeping, or from the expectation-based mechanism, which emphasizes the role of promises in changing trustees’ second-order belief (or both). Public promises play a dual role of activating trustees’ internalized norm of promise-keeping and changing their second-order belief; thus, simply comparing the return level of trustees in the *public promise* treatment with that in the *no promise* treatment cannot differentiate the two influence mechanisms. We attempt to solve this problem by setting up two forms of promises, namely, *self-promise* and *public promise*. In the *self-promise* treatment (T_2_), trustee B makes a promise which is unknown to trustor A; thus, in this case, making a promise activates trustees’ internalized norm of promise-keeping without changing trustors’ first-order belief and trustees’ second-order belief. In the *public promise* treatment (T_3_), trustee B makes a promise to trustor A, which changes trustees’ belief about the expectation of the corresponding trustor due to the latter’s awareness of the promise while activating trustees’ internalized norm of promise-keeping. Along with the aforementioned ideas, we conduct a cross-treatment comparison of individuals’ return behavior and propose the following hypotheses:

**Hypothesis 1** (internalized norm mechanism only): The return level of trustees in the *self-promise* treatment (T_2_) is significantly higher than that in the *no promise* treatment (T_1_), and there is no significant difference between the return level in the *public promise* treatment (T_3_) and that in the *self-promise* treatment (T_2_).

**Hypothesis 2** (expectation-based mechanism only): The return level of trustees in the *public promise* treatment (T_3_) is significantly higher than that in the *no promise* treatment (T_1_), and there is no significant difference between the return level in the *self-promise* treatment (T_2_) and that in the *no promise* treatment (T_1_).

**Hypothesis 3** (both mechanisms): The return level of trustees in the *self-promise* treatment (T_2_) is significantly higher than that in the *no promise* treatment (T_1_), and meanwhile the return level in the *public promise* treatment (T_3_) is significantly higher than that in the *self-promise* treatment (T_2_).

### Procedures

Upon arrival at the laboratory, each participant randomly drew an experimental instruction with a computer number on it and was then seated before a computer with the corresponding number. A pen and some scratch paper had been prepared at each seat for the participants to make calculations during the experimental process. The participants were not allowed to talk with each other until the experiment ended. After all the participants were seated, the experimenter read the instructions aloud. Questions were answered privately. Once all the participants had understood the instructions, namely, no more questions were asked, the experiment started.

Our experiment consisted of four treatments. The participants firstly entered the *preference identification* treatment (T_0_), which comprised only one round. Then, the participants entered the *no promise* treatment (T_1_), *self-promise* treatment (T_2_), and *public promise* treatment (T_3_), in sequence. Each of the latter three treatments comprised two rounds. In each treatment, the program would randomly rematch two participants as a new group.

All aspects of the experiment were common information to the participants. Additionally, the experimental process was completely anonymous, and participants did not know exactly who the other individual was in their group, and their decisions or payoffs were unknown to the others; thus, participants had no concern for the loss of reputation.

After the participants had made all the decisions, they were paid privately in cash and then exited the laboratory. Finally, the experiment ended.

## Results

The summary statistics of the *no promise* treatment, *self-promise* treatment, and *public promise* treatment are presented in Table 2. Overall, participants on average transfer more than half of their endowment (100) to the trustee when playing the role of the trustor. Thereinto, the average amount transferred in the *no promise* treatment and *self-promise* treatment are almost the same, both a little higher than 50, whereas the average amount transferred in the *public promise* treatment is relatively higher, approximately 60, demonstrating that trustors’ awareness of the promise makes a difference to their expectation about the return level of the trustee and, thus, improves trustors’ level of trust. In all the three treatments, the average amount participants return when playing the role of the trustee is higher than the average amount of the transfer in that treatment, that is to say, as a whole, the trust of the trustors is all repaid. In addition, overall, the introduction of promises increases the amount that trustees return, Ret| T_3_ > Ret| T_2_ > Ret| T_1_. In this regard, the main task in the remainder of this paper is to test whether the effect of promises on trustees’ return decision derives from the internalized norm mechanism or the expectation-based mechanism (or both).

**TABLE 2:** Summary statistics of T_1_, T_2_, and T_3__._

	T_1_ (*n* = 60)		T_2_ (*n* = 60)		T_3_ (*n* = 60)	
	Tran| T_1_^a^	Ret| T_1_^b^	Tran| T_2_	Ret| T_2_	Tran| T_3_	Ret| T_3_
Mean	50.85	54.30	51.42	60.12	58.58	65.03
Median	50.00	57.00	50.50	60.00	60.00	55.00
SD	29.01	45.50	31.83	48.75	37.85	54.75

*^a^Tran| T_1_, Tran| T_2_, and Tran| T_3_ represents the amount transferred by trustors in the no promise treatment, self-promise treatment, and public promise treatment, respectively. ^b^Ret| T_1_, Ret| T_2_, and Ret| T_3_ represents the amount returned by trustees in the no promise treatment, self-promise treatment, and public promise treatment, respectively.*

### Mechanism Analysis of the Effect of Promises

Similar to Schwartz et al. (2019), we first analyze trustworthiness to investigate the effect of promises and evaluate whether the internalized norm mechanism or the expectation-based mechanism or both is explanatory for the effect. We use two methods to measure trustworthiness. The first method is directly analyzing the amount returned by trustees: a higher amount represents a higher level of trustworthiness. The second method is analyzing the difference between the amount returned by trustees and the amount the corresponding trustor transferred, which represents the extent to which trustees have repaid the counterparty’s trust: a higher difference represents a higher level of trustworthiness.

We use the paired *t* test to make a cross-treatment comparison of the mean level of participants’ trustworthiness and use the Wilcoxon signed ranks test to estimate whether the distribution of trustees’ return behavior differs significantly between treatments. Through the Shapiro–Wilk test, we find that the difference between trustees’ return decisions in two different treatments is not normally distributed (*p* < 0.05). Thus, we also employ the Wilcoxon signed ranks test to assess if trustees’ return behavior differs significantly between treatments^8^. Table 3 summarizes the results. It should be noted that in the *no promise* treatment, three participants transferred zero when playing the role of trustor A; therefore, there are 57 observations of the “amount returned” and “amount returned–amount transferred” in this treatment. Similarly, in the *self-promise* treatment, two participants transferred zero when playing the role of trustor A, thus there are 58 observations of the “amount returned” and “amount returned–amount transferred” in this treatment. In the *public promise* treatment, six participants transferred zero; thus, observations of the two indicators in this treatment are 54. Therefore, when making cross-treatment comparisons, the number of observations in Table 3 is 56, 52, and 52, respectively, for T_1_ vs. T_2_, T_1_ vs. T_3_, and T_2_ vs. T_3_.

**TABLE 3:** Cross-treatment comparison of trustworthiness.

Treatment	N	Amount returned				Amount returned–Amount transferred			
		Mean	SD	*t* (*p*)	*Z* (*p*)	Mean	SD	*t* (*p*)	*Z* (*p*)
T_1_	56	57.64	45.15	−1.90**^a^ (0.03)	−1.51* (0.07)	3.70	29.45	−2.66*** (0.01)	−2.35*** (0.01)
T_2_		63.84	48.22			10.89	22.13		
T_1_	52	59.13	42.33	−2.12** (0.02)	−2.33** (0.01)	4.33	28.23	−0.41 (0.34)	−0.74 (0.23)
T_3_		69.46	51.49			5.71	34.86		
T_2_	52	65.98	46.47	−0.82 (0.21)	−1.08 (0.14)	11.29	22.20	1.10 (0.14)	−0.66 (0.26)
T_3_		70.62	53.29			6.87	36.31		

^a^ *, **, and *** denote significance at p < 0.10, p < 0.05, and p < 0.01, respectively, for the one-tailed test.

Considering the amount returned by trustees, Table 3 shows that the mean amount returned in the *self-promise* treatment is significantly higher than that in the *no promise* treatment (T_1_ vs. T_2_, *t* stat = −1.90, *p* < 0.05, one-tailed). In the *self-promise* treatment, trustees make a promise which is unknown to the trustor. In this case, making a promise changes neither the trustor’s nor the trustee’s belief. Therefore, the result that *self-promise* significantly increases the amount trustees return is attributed to the activation of their internalized norm of promise-keeping. This result supports the internalized norm mechanism.

Additionally, the mean amount returned by trustees in the *public promise* treatment is also significantly higher than that in the *no promise* treatment (T_1_ vs. T_3_, *t* stat = −2.12, *p* < 0.05, one-tailed). However, there is no significant difference in the mean amount returned between the *public promise* treatment and the *self-promise* treatment (T_2_ vs. T_3_, *t* stat = −0.82, *p* > 0.1, one-tailed; *Z* stat = −1.08, *p* > 0.1, one-tailed). These results indicate no significant difference between the effect of *public promise*, which plays the role of both activating trustees’ internalized norm of promise-keeping and changing their second-order beliefs, and the effect of *self-promise*, which only activates trustees’ internalized norm of promise-keeping. That is, the change of second-order beliefs has no significant effect on the return behavior of trustees, which is not consistent with the expectation-based mechanism. According to the aforementioned analysis of trustworthiness measured by the amount returned, the introduction of *self-promise* or *public promise* makes trustees more trustworthy compared with the case with no promise, and the effect of *self-promise* and *public promise* do not differ significantly. This finding supports Hypothesis 1, that is, we only find evidence supportive of the internalized norm mechanism.

Next, we consider another measurement of trustworthiness: the difference between the amount returned by trustees and the amount transferred by the corresponding trustor, that is, the repayment level of trustees for the counterparty’s trust. In Table 3, the average repayment level of trustees in the three treatments are all above zero, and the average repayment level is the lowest in the *no promise* treatment and the highest in the *self-promise* treatment. Through cross-treatment comparison, we observe that the repayment level of trustees in the *self-promise* treatment is significantly higher than that in the *no promise* treatment (T_1_ vs. T_2_, *t* stat = −2.66, *p* < 0.01, one-tailed; *Z* stat = −2.35, *p* < 0.01, one-tailed), whereas the repayment level in the *public promise* treatment only increases a little compared with that in the *no promise* treatment (T_1_ vs. T_3_). Additionally, no significant difference is observed in the repayment level between the *self-promise* treatment and the *public promise* treatment (T_2_ vs. T_3_). These results are only in line with the internalized norm mechanism for the effect of promises on trustees’ trustworthiness rather than with the expectation-based mechanism, again supporting Hypothesis 1.

Thus far, we have found strong evidence that supports the internalized norm mechanism for promise-keeping through the analyses of participants’ trustworthiness. However, we have not directly investigated the degree to which trustees have kept their promises. Thus, we further compare the promise-keeping level of trustees in the two circumstances with promises. We measure trustees’ promise-keeping level by the difference between the amount they actually return and the amount they have promised to return. A negative difference indicates that the trustee has broken his or her promise, a difference equal to zero indicates that the trustee has just kept his or her promise, and a positive difference indicates that the trustee has kept his or her promise in excess. That is, a higher difference represents a higher level of promise-keeping. The results are presented in Table 4. It should be pointed out that in the *self-promise* treatment, four trustees promised to return 0 in response to the non-zero transfer of the corresponding trustor; thus, these participants are not included in the analysis of trustees’ promise-keeping level. Combined with the condition where trustor A transferred 0, there are 54 observations of the “Amount returned–Amount promised to be returned” in the *self-promise* treatment. Similarly, there are 54 observations of the “Amount returned–Amount promised to be returned” in the *public promise* treatment. Therefore, the number of observations in Table 4 is 49 when making a cross-treatment comparison of trustees’ promise-keeping level between the *self-promise* treatment and the *public promise* treatment.

**TABLE 4:** Cross-treatment comparison of the promise-keeping level.

	Amount returned–Amount promised to be returned	
	T_2_	T_3_
Mean	−3.16	−11.41
SD	18.05	28.48
*t* (*p*)	1.94 (0.03)**^a^	
*Z* (*p*)	−1.73 (0.04)**	
*N*	49	
#≥0^b^	26/49	26/49

^a^ ** denotes significance at p < 0.05 for the one-tailed test. ^b^ #≥0 denotes the proportion of trustees for whom the difference between the amount returned and the amount promised to be returned is greater than or equal to 0.

The results reported in Table 4 show that the proportion of trustees who keep their promise (the difference between the amount actually returned and the amount promised to be returned is greater than or equal to zero) in the *self-promise* treatment is the same as that in the *public promise* treatment (26/49, 53%). Overall, the mean amount actually returned by trustees is lower than the mean amount promised to be returned in the two treatments. Specifically, the difference between the mean amount actually returned and the mean amount promised to be returned by trustees is −3.16 in the *self-promise* treatment and −11.41 in the *public promise* treatment. In addition, the difference in the promise-keeping level of trustees between the *self-promise* treatment and the *public promise* treatment is significant at one-tailed *p* < 0.05. In other words, the mean level of promise-keeping does not improve from the introduction of *public promises* but, by contrast, is significantly lower than that in the *self-promise* treatment, demonstrating that the activation of participants’ internalized norm of promise-keeping rather than their second-order belief about the counterparty’s expectation significantly influences their promise-keeping level. This finding does not lend support to Hypothesis 2, the expectation-based mechanism.

Notably, no significant difference is observed in the level of trustworthiness between the *public promise* treatment and the *self-promise* treatment, and trustees’ promise-keeping level in the *public promise* treatment is significantly lower than that in the *self-promise* treatment. The possible explanation for these findings is as follows. In the *public promise* treatment, trustees first make their promises, and then trustors decide the amount to transfer after observing the corresponding trustee’s promise. In this case, trustees’ promises may influence the amount they receive later; thus, out of strategic consideration, they are likely to make an unreal promise, which is excessively high, to elicit a greater amount transferred from the trustor, actually generating a relatively lower level of promise-keeping in the *public promise* treatment.

Based on the aforementioned analyses, we find evidence consistent with the internalized norm mechanism, and we find no support for the expectation-based mechanism. Therefore, our experimental results are in line with those of Ismayilov and Potters (2016) but contrast with those of Schwartz et al. (2019). Specifically, the experimental results of Ismayilov and Potters (2016) suggest that promises increase trustworthiness even when messages are not delivered, supporting the internalized norm mechanism for promise-keeping. Thus, our conclusion is in accordance with theirs after controlling for the self-selection effect that exists in their experimental results. However, in the experiment of Schwartz et al. (2019), only delivered promises increase trustworthiness, and undelivered promises produce almost the same behavior as non-promises, which is inconsistent with the internalized norm mechanism and supports only the expectation-based mechanism for promise-keeping.

### Heterogeneity Analysis

On the basis of the aforementioned results, we attempt to incorporate heterogeneity of individuals’ other-regarding preferences into our analyses to investigate the difference in the effect of promises between individuals with heterogeneous preferences and further assess the empirical support for the internalized norm mechanism and the expectation-based mechanism. In the *preference identification* treatment, we make all the participants trustee B and ask them to decide how many tokens they are willing to return as per all the possible amounts trustor A may transfer (integers in the interval [1, 20]), which is tripled on the way to trustee B. By this means, we can observe how participants adjust the amount they return according to the amount the other party transfers and then identify the type of their preferences.

Specifically, we conduct a linear regression with the amount conditionally returned by trustees as dependent variables and integers in the interval [1, 20] as independent variables, generating a slope *k* for each participant. Then, we can estimate the type of their preferences according to the size of their slopes. We classify the participants with *k* ≥ 1 as conditional cooperators. For this type of participants, the amount they return when playing the role of the trustee increases with the amount transferred by the trustor, and the former is mainly greater than or equal to the latter. The participants with *k* < 1 are classified as selfish individuals. In our experiment, there are 22 selfish participants and 38 conditional cooperators. Four of the 38 conditional cooperators are perfect conditional cooperators with *k* = 1, who return exactly the amount the counterparty transfers. In the remainder of this section, we conduct a cross-treatment comparison of behavior for these two types of trustees, respectively. The results are presented in Table 5. The number of observations, *N*, in Table 5 can be explained by the same means used to calculate the number of observations in Table 3.

**TABLE 5:** Cross-treatment comparison of trustworthiness for selfish participants and conditional cooperators.

Type	Treatment	N	Amount returned				Amount returned–Amount transferred			
			Mean	SD	*t* (*p*)	*Z* (*p*)	Mean	SD	*t* (*p*)	*Z* (*p*)
Selfish participants	T_1_	21	30.71	48.14	0.29 (0.39)	−0.96 (0.17)	−11.71	32.11	−1.85** (0.04)	−1.59* (0.06)
	T_2_		29.38	48.54			−1.86	20.10		
	T_1_	16	28.88	42.39	−1.99**^a^ (0.03)	−2.03** (0.02)	−14.25	29.89	0.77 (0.23)	−0.42 (0.34)
	T_3_		37.56	47.70			−20.63	38.86		
	T_2_	16	29.44	46.02	−1.24 (0.12)	−1.61* (0.05)	−2.75	19.80	2.32** (0.02)	−1.82** (0.03)
	T_3_		37.56	47.70			−20.63	38.86		
Conditional cooperators	T_1_	35	73.80	34.89	−2.49*** (0.01)	−2.26** (0.01)	12.94	23.70	−1.89** (0.03)	−1.72** (0.04)
	T_2_		84.51	34.68			18.54	19.85		
	T_1_	36	72.58	35.16	−1.62* (0.06)	−1.71** (0.04)	12.58	23.46	−1.50* (0.07)	−1.41* (0.08)
	T_3_		83.64	47.08			17.42	25.81		
	T_2_	36	82.22	36.84	−0.40 (0.34)	−0.45 (0.33)	17.53	20.49	−0.35 (0.36)	−0.64 (0.26)
	T_3_		85.31	49.41			19.08	27.84		

^a^ *, **, and *** denote significance at p < 0.10, p < 0.05, and p < 0.01, respectively, for the one-tailed test.

We first examine the effect of *self-promise* and *public promise* on the trustworthiness of selfish individuals to assess the empirical support for the internalized norm mechanism and the expectation-based mechanism for this type of participants’ behavior. As shown in Table 5, in terms of the amount returned, selfish trustees significantly return more on average in the *public promise* treatment than in the *no promise* treatment, and no significant difference is observed in the amount returned between the *self-promise* treatment and *no promise* treatment. These results seem to be inconsistent with the internalized norm mechanism and support only the expectation-based mechanism. However, as aforementioned, in the *public promise* treatment, trustees are likely to make an excessively high promise, which they are not going to keep, to elicit a greater amount transferred from the trustor. Since selfish individuals attach more importance to the monetary payoff they receive, they are more likely to take such strategic considerations into their decision-making. Additionally, after observing trustees’ high promises, the corresponding trustor may increase the amount to transfer, which may generate a relatively higher amount returned by selfish trustees in the *public promise* treatment. But actually, the extent to which selfish trustees have repaid the counterparty’s trust may, by contrast, be even lower in the *public promise* treatment. Therefore, for selfish individuals, conclusions based on the analyses of the amount they return alone may be biased.

We subsequently investigate the repayment level of selfish trustees, which is measured by the difference between the amount returned by trustees and the amount transferred by the corresponding trustor. The results in Table 5 show that the mean amount returned by selfish trustees is lower than the mean amount transferred by trustors in all the three treatments. That is, the repayment level of selfish trustees is negative in each treatment; thereinto, the repayment level in the *self-promise* treatment is the highest, and the repayment level in the *public promise* treatment is the lowest of the three. These results also confirm our conjecture in the previous paragraph: in the *public promise* treatment, selfish trustees are prone to make an excessively high promise to elicit a greater amount transferred from the trustor, which indeed helps them achieve a greater amount of tokens; thus, in this treatment, selfish trustees may seemingly return more than in the other two treatments but their actual level of trustworthiness is the lowest in the *public promise* treatment.

In Table 5, the repayment level of selfish trustees in the *public promise* treatment is significantly lower than that in the *self-promise* treatment (T_2_ vs. T_3_, *t* stat = 2.32, *p* < 0.05, one-tailed; *Z* stat = −1.82, *p* < 0.05, one-tailed), again proving the negative effect of strategic consideration on the trustworthiness of selfish participants in the *public promise* treatment. Additionally, their repayment level in the *self-promise* treatment is significantly higher than that in the *no promise* treatment (T_1_ vs. T_2_, *t* stat = −1.85, *p* < 0.05, one-tailed). This finding suggests that although selfish individuals attach more importance to their monetary payoffs (manifested by the negativity of their repayment level), the impact of internalized norms remains significant, even if small in magnitude. That is, making a promise activates their internalized norm of promise-keeping, and they may experience disutility due to any deviation from the norm and, thus, be inclined to cooperate. Additionally, the repayment level of selfish trustees in the *public promise* treatment is slightly lower than that in the *no promise* treatment (T_1_ vs. T_3_), which is consistent with the above findings. That is, in the *public promise* treatment, the positive effect of the norm of promise-keeping on selfish participants’ trustworthiness is offset by the negative effect of strategic consideration, generating a similar level of trustworthiness in this treatment with that in the *no promise* treatment. In summary, findings from the analyses of selfish participants’ behavior are only consistent with the internalized norm mechanism and still not in support of the expectation-based mechanism for the effect of promises.

Next, we examine the effect of promises on conditional cooperators’ trustworthiness. The results reported in Table 5 suggest that the amount returned by conditional cooperators in the *self-promise* treatment is significantly higher than that in the *no promise* treatment (T_1_ vs. T_2_, *t* stat = −2.49, *p* < 0.01, one-tailed; *Z* stat = −2.26, *p* < 0.05, one-tailed), indicating that the activation of the internalized norm of promise-keeping plays a critical role in improving the return level of conditional cooperators. In addition, the mean amount returned by conditional cooperators in the *public promise* treatment is only marginally higher than that in the *no promise* treatment, but the distribution of their return behavior is significantly different between the two treatments (T_1_ vs. T_3_, *Z* stat = −1.71, *p* < 0.05, one-tailed). Additionally, there is no significant difference in the amount returned by this type of trustees between the *public promise* treatment and the *self-promise* treatment (T_2_ vs. T_3_). These findings suggest that changes in conditional cooperators’ second-order beliefs have no significant impact on the amount they return, which is still inconsistent with the expectation-based mechanism.

In terms of the repayment level of conditional cooperators, the results in Table 5 show that compared with the *no promise* treatment, the introduction of *self-promises* has a significant effect on the repayment level of conditional cooperative trustees (T_1_ vs. T_2_, *t* stat = −1.89, *p* < 0.05, one-tailed; *Z* stat = −1.72, *p* < 0.05, one-tailed), and the introduction of *public promises* only has a marginal effect (T_1_ vs. T_3_). Additionally, no significant difference is observed in their repayment level between the *self-promise* treatment and the *public promise* treatment (T_2_ vs. T_3_). In summary, the results from the analyses of conditional cooperators’ return behavior indicate that the activation of the internalized norm of promise-keeping has a significant effect on improving conditional cooperators’ trustworthiness, and the change in their second-order beliefs does not. These results support only the internalized norm mechanism (Hypothesis 1).

Finally, the results from a cross-treatment comparison of these two types of participants’ promise-keeping level (denoted by the difference between the amount returned and the amount promised to be returned by trustees) still cannot support the expectation-based mechanism. Specifically, for selfish participants, their mean level of promise-keeping is −3.77 in the *self-promise* treatment and −19.05 in the *public promise* treatment; and for conditional cooperators, their mean level of promise-keeping is −2.94 in the *self-promise* treatment and −8.66 in the *public promise* treatment. These results suggest that the promise-keeping level in the *public promise* treatment is lower than that in the *self-promise* treatment for both selfish trustees and conditional cooperative trustees, that is, the increase of these two types of participants’ second-order beliefs has no effect in improving their promise-keeping level, which is inconsistent the expectation-based mechanism for the effect of promises.

## Discussion

According to the assumption of *homo economicus*, cooperation among self-interested individuals is maintained through external reward and punishment institutions and reputation incentives arising from repeated interactions; verbal promises and threats among anonymous individuals are not credible. However, many experimental studies find that non-binding verbal promises have profound effects on cooperation, even in strictly one-shot settings. It is important to understand what motivates an individual to keep his or her promise in the absence of external punishment or reputational concern.

Two leading influence mechanisms have been proposed. The first one is the internalized norm mechanism, which assumes that making promises activates promisors’ internalized norm of promise-keeping and the one suffers a cost from breaking his or her promise *per se*. The second one is the expectation-based mechanism, which assumes that a promise may change the promisee’s expectation about the outcome and the promisor keeps his or her promise to avoid the feeling of guilt from letting down the other party. However, there is still much debate regarding the validity of the two influence mechanisms for the effect of promises in promoting cooperation. Empirical evidence of the two mechanisms seems confounded.

The aim of this paper is then to propose a new experimental design to distinguish the internalized norm mechanism from the expectation-based mechanism and provide reliable experimental evidence for the two mechanisms.

We adopted a within-subject setting and designed three separate treatments, namely, the *no promise* treatment, *self-promise* treatment, and *public promise* treatment, based on the trust game of Berg et al. (1995), to conduct a cross-treatment comparison of the same trustees’ return behavior and investigate the effect of promises on it and the underlying mechanism of the effect. Additionally, considering the effect of heterogeneous preferences on individual behavior, we designed a *preference identification* treatment to determine the type of each participant’s cooperation preference to further investigate whether the effect of promises and its underlying mechanism would differ among individuals with different preferences. Our experimental results show the following:

First, overall, the return level of trustees in the *self-promise* treatment is significantly higher than that in the *no promise* treatment, and no significant difference is observed in the return level between the *public promise* treatment and the *self-promise* treatment, which supports only the internalized norm mechanism;

Then, for selfish individuals, their mean return level is relatively low, but their return level in the *self-promise* treatment is significantly higher than that in the *no promise* treatment, which supports the internalized norm mechanism for the effect of promises on selfish individuals’ return behavior, and their return level in the *public promise* treatment is instead slightly lower than that in the *no promise* treatment, which cannot be interpreted by the expectation-based mechanism;

Finally, for conditional cooperators, their return level in the *self-promise* treatment is significantly higher than that in the *no promise* treatment, and there is no significant difference in their return level between the *public promise* treatment and the *self-promise* treatment. This finding still supports only the internalized norm mechanism.

In summary, our experimental results provide strong empirical evidence that supports the internalized norm mechanism, that is, making a promise activates the internalized norm of promise-keeping within individuals, and any deviation from the norm *per se* may cause disutility for them even when their promise is unknown to the promisee.

The results we obtain in this paper cannot be interpreted by the expectation-based mechanism. However, this does not mean that the two influence mechanisms of promises are mutually exclusive, since an individual may be inclined both to keep his or her promise and to fulfill the expectation created by the promise to avoid the feeling of guilt. Different individuals might be predominantly motivated by different concerns when making decisions in a given context. The inclination to conform to the norm (i.e., promise-keeping) activated by certain contextual signals varies across individuals (Ostrom, 2000; Fischer and Huddart, 2008; Kessler and Leider, 2012); an individual with higher inclination will experience more disutility if his or her behavior deviates from the legitimate action in the context. The sensitivity degree to the feeling of guilt also varies across individuals; an individual with a higher sensitivity degree will suffer more disutility if his or her action leads to an outcome that may disappoint the other party. In this paper, we only provide empirical evidence for the two influence factors, and we cannot measure the specific weight each individual attaches on the two factors when making decisions. We will attempt to address this limitation with a follow-up study that mixes within-subject and between-subject design supplementing the experimental paradigm offered in this paper.

Based on our methods and results, we also suggest the following topics for further investigation. For the simplicity of our experimental settings, we did not measure the beliefs of trustors or trustees in our experiment. Under this circumstance, we cannot further confirm whether participants have strategic considerations during the experimental process. In addition, regarding the impact of belief elicitation on subjects’ decisions, Guerra and Zizzo (2004) argue that there is no significant impact, whereas Ockenfels and Werner (2014) demonstrate that belief elicitation may make a subtle difference to individual behavior. Therefore, in further experiments, we could attempt to incorporate interacting parties’ beliefs into the analysis while controlling the interference factors in the process of belief elicitation as much as possible to make more explicit the internal motivations of individual behavior.

In addition, in the experiments conducted by Ismayilov and Potters (2016) and Schwartz et al. (2019), trustors’ decision is a binary variable (cooperate or not cooperate), and trustees make their decisions under the assumption that the trustor chooses to cooperate. By contrast, in our experiment, trustors’ trusting behavior is a continuous variable, and trustees’ decision may also be affected by the corresponding trustor’s choice, especially in the *public promise* treatment, where the effect of promises on trustees’ behavior results from the joint effect of the promise *per se* and the trustor’s choice affected by the promise, thus possibly impacting the identification of the influence mechanism of promises. Hence, in further experiments, we could attempt to isolate the influence of the counterparty’s behavior when investigating the effect of promises on the behavior of the promisor.

In our experiment, promises are voluntarily made by each trustee. Some researchers have asserted that promises elicited by a third party (e.g., the experimenter) are not as effective as voluntary promises in promoting promisors’ cooperation level (Belot et al., 2010; Charness and Dufwenberg, 2010), whereas promises elicited by the corresponding trustor play a similar role in promoting cooperation as voluntary promises (Ismayilov and Potters, 2017). In subsequent researches, we could attempt to investigate the aforementioned three forms of promises within a uniform experimental structure to explore the different effects of voluntary promises, third-party-elicited promises, and trustor-elicited promises on promisors’ cooperation behavior and explain the underlying mechanism of this difference on the basis of the conclusions of this paper.

Finally, although the work in our paper is not related to neuroscience, it will be illuminating if we can propose a model connecting behavior with the brain (Di Plinio and Ebisch, 2020) and relate our experimental findings here to some neuronal findings. In this work, we assume that an individual keeps his or her promise to avoid the negative feelings like guilt (arising from not fulfilling the promisee’s expectation about the outcome), shame or anxiety (arising from breaking the norm of promise-keeping), which is closely correlated with spontaneous brain activity. Researches in neuroscience have found that spontaneous brain activity contributes to a predisposition for social behavior with external stimuli. How brain activities differ in our three main treatments? An investigation about this question will further help us understand the intrinsic mechanisms underlying the effect of promises. Hence, we may attempt to establish a behavioral-brain connection in further researches.

## Data Availability Statement

The raw data supporting the conclusions of this article will be made available by the authors, without undue reservation.

## Ethics Statement

The studies involving human participants were reviewed and approved by the Research Ethics Committee of the School of Economics and Management, Nanjing University of Science and Technology. The patients/participants provided their written informed consent to participate in this study.

## Author Contributions

XZ conceived and designed the experiments. WZ and HG performed the experiments and wrote the first draft of the manuscript. WZ, HG, XZ, and TL participated in the data analysis and data interpretation. All authors contributed to manuscript revision, read and approved the submitted version.

## Conflict of Interest

The authors declare that the research was conducted in the absence of any commercial or financial relationships that could be construed as a potential conflict of interest.

## Footnotes

1 Vanberg (2008) refers to the internalized norm mechanism in our paper as the commitment-based explanation.

2 In the communication stage, trustees can write a free-form message to trustors. Results in the literature have shown that most trustees send messages containing promises such as “If you cooperate, I will cooperate too,” and other trustees may send an empty talk or no message.

3 Both delivered messages and undelivered messages comprise messages containing promises and messages not containing promises (empty talk or no message).

4 It is worth noting that researches in psychology and neuroscience have found that spontaneous brain activity, which is intrinsically related to the self and the interaction with stimuli (Legrand and Ruby, 2009; Sui and Humphreys, 2015; Northoff, 2016; Scalabrini et al., 2018, 2020), contributes to a predisposition for social behavior and this may be modulated by a personality like narcissism which is featured by the continuum of selfishness and disconnection with the others (Brown et al., 2010; Newton et al., 2014; Scalabrini et al., 2017; Krizan and Herlache, 2018). Our work in this paper is to test the internalized norm mechanism, which is part of individuals’ self-organization, and the expectation-based mechanism, which is related to the contextual fear of hurting other’s feelings. We also test whether the effectiveness of the two mechanisms differs between selfish individuals and conditional cooperators. It appears that these two kinds of work are similar in some ways. We may refer to the studies in psychology and neuroscience to enlarge our theoretical background in future researches.

5 In the *self-promise* treatment, trustor A is endowed with 100 tokens. Thus, we ask trustee B to promise the ratio of tokens he or she is willing to return as per all the possible intervals that the transferred amount by trustor A may lie in (i.e., [0, 10); [10, 20);… [80, 90); [90, 100]). As the amount received by trustee B is tripled, the ratio r trustee B promises to return is restricted in the interval [0, 3], and *r* = 1 means that the trust of trustor A is just repaid.

6 Our experimental setup only prescribes a limit to the form of promises, and the content of promises is still determined by the participants, not exogenously given. See Lundquist et al. (2009) and Charness and Dufwenberg (2010) for the difference between the effect of exogenously fixed promises and endogenously determined promises.

7 We use the independent-samples *t* test to examine if there is a difference in the return level between trustees who play the role of trustee B in the first round and trustees who play the role of trustee B in the second round in the three treatments. We find no significant difference between the return level of the two kinds of trustees in each of the three treatments (*t* = 0.732, *p* = 0.467, two-tailed, in the *no promise* treatment; *t* = −0.030, *p* = 0.976, two-tailed, in the *self-promise* treatment; *t* = −0.623, *p* = 0.536, two-tailed, in the *public promise* treatment). It seems that there is no order effect of roles. Therefore, we do not distinguish between these two kinds of trustees in the following analyses.

8 Many researches regarding the effect of promises refer to the binary trust game of Charness and Dufwenberg (2006), in which trustees’ behavior is binary. Schwartz et al. (2019) extend the binary decision-making of trustees to trustees’ return decision, which is in the form of consecutive integers. Our experimental design is closely relevant to that of Schwartz et al. (2019). In their paper, they also conduct both a paired *t* test and a Wilcoxon signed ranks test to assess the difference between delivered and undelivered promises. Therefore, our experimental results are comparable to the results of Schwartz et al. (2019).
