# Supplementary material for: Self-Conscious or Fear of Hurting Another’s Feeling? An Experimental Investigation on Promise-Keeping
Source: Front Psychol. 2020 Sep 30;11:576824. doi: 10.3389/fpsyg.2020.576824 (PMC7561381; doi:10.3389/fpsyg.2020.576824)
Supplement: Supplementary file 1 [file Table_1.DOC]

Supplementary Material

**Instructions**

Thank you for participating in our experiment. Please read the instructions carefully and complete the experiment seriously.

In the experiment, you gain experimental tokens through a series of decision-tasks, and the amount of tokens you gain depends on your actions and others’. We will cash the experimental tokens in a certain ratio at the end of the experiment. In addition, every participant will receive RMB 5 for participating in this experiment.

Your decisions and other personal information will not be shared with anyone. Please complete the experiment according to the instructions on the computer interface. You are not allowed to communicate with other participants during the experiment. If you have any questions, please raise your hand and ask the experimenters only. Thank you for your cooperation. Please read the following detailed instructions.

This is an investment experiment. The experimental structure is as follows.

Two individuals will be randomly matched into a group, and each group can receive S tokens as initial wealth. Randomly selected one of the two members will have the role of A, and the other member will have the role of B.

*Step 1*: Player A decides to transfer y tokens, which is restricted in the interval [0, S], to Player B. The experimental program triples the amount transferred to 3y on the way to Player B.

*Step 2*: Player B decides to return x tokens, which is restricted in the interval [0, 3y], to A.

The final payoff of Player A and B is (S–y+x) and (3y–x), respectively.

The following experiment comprises four different stages based on the explained experimental structure. Please read the instructions below carefully.

**Stage 1:**

You are randomly matched with another participant as a group, and your group has 20 tokens. The experimental program does not assign roles within your group at the beginning. You need to complete the decision-tasks of Player A and Player B, respectively, and then, the program will randomly decide your role and calculate your payoff based on the decisions you and your partner has made for the role you are actually assigned.

Step 1: As Player B, you need to fill in the following table, which lists all the possible amounts Player A can transfer (1-20). Please decide how many tokens to return as per all the possible amounts Player A may transfer after receiving 3 times the amount Player A transfers:

| A transfers | 1 | 2 | 3 | 4 | 5 | 6 | 7 | 8 | 9 | 10 | 11 | 12 | 13 | 14 | 15 | 16 | 17 | 18 | 19 | 20 |
| --- | --- | --- | --- | --- | --- | --- | --- | --- | --- | --- | --- | --- | --- | --- | --- | --- | --- | --- | --- | --- |
| B returns |  |  |  |  |  |  |  |  |  |  |  |  |  |  |  |  |  |  |  |  |

*Step 2*: As Player A, you decide to transfer __ (0-20) tokens to Player B.

*Step 3*: The computer program randomly assigns roles and calculates your payoff as the role you are actually assigned.

*Payoff calculation*: If you are assigned the role of A, your payoff is (20–the amount you transfer+the amount returned by the corresponding B). If you are assigned the role of B, your payoff is (3*the amount transferred by the corresponding A–the amount you return as indicated in the table); for example, if Player A transfers 15 to you and you decide to return R conditional on A transferring 15 in the table, your payoff is (3*15–R).

**Stage 2:**

You are randomly matched with another participant as a group and assigned the role of Player A or Player B, and your group has 100 tokens. There are two rounds in this stage:

Round 1: Player A decides how many tokens to transfer. Next, Player B decides how many tokens to return after receiving 3 times the amount A transfers.

Round 2: The computer program randomly rematches two players as a new group, assigns roles, and repeats the procedure of Round 1.

The program randomly selects one of the two rounds to determine the payoffs of this stage.

**Stage 3:**

You are randomly matched with another participant as a group and assigned the role of Player A or Player B, and your group has 100 tokens. There are two rounds in this stage:

Round 1:

Step 1: Player B makes a **‘self-promise’** about the proportion of tokens he or she is willing to return (0-3) as per all the possible amounts Player A may transfer after receiving 3 times the amount Player A transfers:

| A transfers k to you | k | 0-10 | 10-20 | 20-30 | 30-40 | 40-50 | 50-60 | 60-70 | 70-80 | 80-90 | 90-100 |
| --- | --- | --- | --- | --- | --- | --- | --- | --- | --- | --- | --- |
| proportion α you are willing to return after receiving 3k | α |  |  |  |  |  |  |  |  |  |  |

Note: **The proportion written here is a promise to yourself, and this table will not be revealed to anyone else.** (You can fill in any two-decimal number, α, that lies in the interval [0, 3]. For example, α=1 means that you just repay the amount A transfers; and α=1.2 means that you return 20% more than the amount A transfers, that is, 1.2*k.)

Step 2: Player A decides how many tokens to transfer.

Step 3: Player B decides how many tokens to return after receiving 3 times the amount A transfers.

Round 2: The computer program randomly rematches two players as a new group, assigns roles, and repeats the procedure of Round 1.

The program randomly selects one of the two rounds to determine the payoffs of this stage.

**Stage 4:**

You are randomly matched with another participant as a group and assigned the role of Player A or Player B, and your group has 100 tokens. There are two rounds in this stage:

Round 1:

Step 1: Player B makes a **‘public promise’ to Player A** about the proportion of tokens he or she is willing to return (0-3) as per all the possible amounts Player A may transfer after receiving 3 times the amount Player A transfers:

| A transfers k to you | k | 0-10 | 10-20 | 20-30 | 30-40 | 40-50 | 50-60 | 60-70 | 70-80 | 80-90 | 90-100 |
| --- | --- | --- | --- | --- | --- | --- | --- | --- | --- | --- | --- |
| proportion α you are willing to return after receiving 3k | α |  |  |  |  |  |  |  |  |  |  |

Note: **The proportion written here will be revealed to the Player A in your group, and Player A decides the amount that will be actually transferred to you after observing the table.** (You can fill in any two-decimal number, α, that lies in the interval [0, 3]. For example, α=1 means that you just repay the amount A transfers; and α=1.2 means that you return 20% more than the amount A transfers, that is, 1.2*k.)

Step 2: Player A decides how many tokens to transfer after observing the promise table of Player B, who is matched with Player A.

Step 3: Player B decides how many tokens to return after receiving 3 times the amount A transfers.

Round 2: The computer program randomly rematches two players as a new group, assigns roles, and repeats the procedure of Round 1.

The program randomly selects one of the two rounds to determine the payoffs of this stage.
